# Measuring Medicine Use: Applying ATC/DDD Methodology to Real-World Data

**DOI:** 10.3390/pharmacy9010060

**Published:** 2021-03-17

**Authors:** Samantha Hollingworth, Therése Kairuz

**Affiliations:** 1 School of Pharmacy, University of Queensland, Woolloongabba, QLD 4102, Australia; s.hollingworth@uq.edu.au; 2 Faculty of Pharmacy and Pharmaceutical Sciences, Kwame Nkrumah University of Science and Technology, Kumasi, Ghana; 3 School of Pharmacy, University of Newcastle, Callaghan, NSW 2308, Australia; 4 Centre for African Research, Engagement and Partnerships (CARE-P), University of Newcastle, Callaghan, NSW 2308, Australia

**Keywords:** medicines, pharmacoepidemiology, ATC, DDD, drug utilization

## Abstract

Medicines are essential for the treatment of acute, communicable, and non-communicable diseases. The World Health Organization developed a toolkit for drug (medicine) utilization studies to assist in reviewing and evaluating the prescribing, dispensing, and use of medicines. There is a growing need for rigorous studies of medicine use in low- and middle-income countries (LMIC) using standard approaches, especially in the context of universal health coverage. This commentary provides a succinct summary of how to use the WHO anatomical therapeutic chemical (ATC)/defined daily dose (DDD) methodology in pharmacoepidemiological studies, with a focus on LMIC contexts. We drew on information from WHO resources and published literature, citing examples and case studies. We encourage readers to publish their drug utilization studies, although we caution about predatory journals. We recommend the use of the RECORD-PE initiative which focuses on methods for doing pharmacoepidemiological research and evaluating the quality of published papers.

## 1. Introduction

Medicines are essential for the treatment of acute, communicable, and non-communicable diseases and medical conditions, and include prescription, over the counter, and complementary and alternative medicines. Prescription medicines—and the associated medical services—are a considerable expense in any country. In many places, but especially in low- and middle-income countries (LMIC), this cost is often borne by the patient but in many high-income countries—and particularly those that have some form of public health system—such costs are heavily subsidized by the government. Many LMICs are working towards universal health coverage (UHC) where “*all people [will] have access to needed health services (including prevention, promotion, treatment, rehabilitation and palliation) of sufficient quality to be effective while also ensuring that the use of these services does not expose the user to financial hardship*” [1].

UHC invariably means developing a national health insurance system covering medical services and medicines for defined populations delivered by hospitals, health clinics, and community pharmacies. It usually includes hospital services for acute and emergency treatment. The government reimburses providers for their services or products. Claims databases and other data sources are often used to study the use of health services and supply of medicines, for example Medicare in Australia [2] and the National Health Insurance Scheme in Ghana [3]. 

Pharmacoepidemiology is the study of the use of medicines, and their risks, benefits, and harms in populations. There is growing interest in pharmacoepidemiological studies in LMIC, including Africa [4]. Such studies provide vital information on the rational use of medicines to ensure that “*patients receive medications appropriate to their clinical needs, in doses that meet their own individual requirements, for an adequate period of time, and at the lowest cost to them and their community*.” [5] Knowing how medicines are used by populations contributes directly to our understanding of, and reaction to, global health threats such as antimicrobial resistance [6] and non-communicable diseases [7]. 

The World Health Organization (WHO) developed a toolkit for drug (medicine) utilization studies to assist in reviewing and evaluating the prescribing, dispensing, and use of medicines [8]. Each medicine is allocated a unique code using the anatomical therapeutic chemical (ATC) classification system, and a defined daily dose (DDD). This universally-accepted methodology comprises letters and numbers and facilitates international, national, and regional comparisons. ATC/DDD is a dynamic system and readers are encouraged to use the current classification regardless of the age of the data [9].

Most of the early medicine use (pharmacoepidemiological) studies have been published from countries in Europe and North America which have administrative databases derived from their UHC. Drug utilization studies in Africa have been actively encouraged by the establishment of Medicines Utilisation Research in Africa (MURIA) in 2015 at the Nelson Mandela University in South Africa [4].

The WHO South-East Asia Region (SEARO) is home to one quarter of the world’s population [10]. Surprisingly, only one in five medicine use studies used the WHO ATC/DDD system, as revealed in a systematic review of studies emanating from that region. To the best of our knowledge, there are no systematic reviews from other regions that include LMICs. The authors concluded that medicine use studies and the use of the ATC/DDD system need to be promoted and conducted in LMIC [10]. Furthermore, anecdotal evidence gained at conferences in Africa suggests that a guide to how to apply ATC/DDD would be useful for researchers who are new to drug utilization studies. Together with the paucity of these studies from Asia, this provided the impetus for the authors to compile this commentary in which we aim to provide a succinct summary of how to use the ATC/DDD methodology in pharmacoepidemiological studies in adult populations, with a focus on LMIC contexts, drawing on information available from the WHO Collaborating Centre for Drug Statistics Methodology and WHO toolkit [8,11]. This commentary includes descriptions and explanations about the ATC classification system and the DDD, as well as how to perform relevant calculations; we also provide examples of sources of data. The information is relevant for studies among adult populations. There are additional considerations for drug utilization studies in children [12,13].

## 2. The ATC Classification System

The ATC/DDD system is a global standard overseen by WHO [14]. Active medical substances—commonly referred to as the active ingredient(s) in a medicine—are grouped according to the organ (e.g., heart, kidney) or body system (e.g., central nervous system) on which they exert their effect. Stated simply, the ATC classification is an alphabetical and numerical descriptor of the properties of an active ingredient (commonly referred to as a “drug” or “medicine”) classified into one of five levels in the ATC system. 

The first level describes one of fourteen anatomical or body systems: alimentary tract and metabolism [A], blood and blood-forming organs [B], cardiovascular [C], dermatologicals [D], genitourinary and sex hormones [G], systemic hormonal preparations excluding sex hormones and insulin [H], general anti-infectives for systemic use [J], antineoplastics and immunomodulating agents [L], musculoskeletal [M], central nervous system [N], antiparasitic [P], respiratory [R], sensory organs [S], and “various” [V] which includes other therapeutic products. 

The second, third, and fourth levels offer descriptions of the therapeutic and pharmacological actions, and the chemical name of the drug. Using atenolol as an example, it is classified as cardiovascular (i.e., body system), followed by the therapeutic class (antihypertensive), the pharmacological action (e.g., beta-blocker), and the chemical descriptor (atenolol), culminating in the ATC code of C07AB03. This system permits international communication about this drug (or active ingredient or medicine), without the complications associated with language and spelling. However, it is important to highlight some anomalies. For example, C02-cardiovascular antihypertensives is a therapeutic label even though it occurs on the same level as some pharmacological labels such as C03-diuretics and C07-beta blocker agents which is confusing for many users. It is important to note that many of the drugs used to treat hypertension are NOT included in the group C02-antihypertensives.

Coding is important in pharmacoepidemiological studies as it promotes accuracy by clearly identifying the medicine; however, ATC/DDD is associated with the dosage form of a drug and therefore there may be more than one ATC (and DDD) for a particular drug. For example, a drug which is available as a tablet and injection would have two different ATC codes and possibly two DDDs. 

Clinicians, researchers, health professionals, and patients most often use the chemical or innovator brand name of a medicine. We note that the term “generic” is often used to describe an active ingredient, although “generic” is the term commonly used to describe a non-innovator product such as diclofenac, for which the branded innovator product is marketed as Voltaren^®^. When a medicine name is used, the official standard international nonproprietary name (INN) is preferred. Two alternatives to the use of INN are the United States adopted name (USAN) and the British approved name (BAN). Compliance with INN is ongoing and there has been a concerted global effort to implement the INN [15]. Australia, for example, is updating the names of 20 active ingredients on the Australian Essential Medicines List at the time of writing this paper; the diuretic frusemide (ATC C03CA01) is one of the drug names that has changed to the INN of furosemide. It is expected that universal use of the INN will minimize prescribing errors. 

## 3. The Defined Daily Dose (DDD)

The DDD is used in conjunction with ATC: ATC describes the drug while the DDD is defined as the average maintenance dose per day for the main indication of the drug in adults, expressed in various units, e.g. milligrams or grams, which may differ by route of administration [11]. The DDD was developed to overcome challenges with dosage forms and is also a convenient way of following changes in use over time especially when the mix of formulations changes or even when there are changes in pack sizes which often occurs in hospitals. The DDD is not to be confused with the prescribed daily dose (PDD) which is defined as the average dose prescribed according to a representative sample of prescriptions. It is important to take into account the condition for which the dose was prescribed. It is important to be aware of the different ATC/DDDs, for example, when calculating PDD/DDD.

As with many calculations of an average, the DDD does not necessarily reflect a recommended dose, especially when a dose needs to be adjusted for the patient, such as among the elderly. Only when the DDD is in close agreement with the PDD will the DDD represent the actual use of the drug as the PDD accounts for disease severity and patient factors such as age, sex, weight, ethnicity, and pharmacokinetics. 

Factors associated with medicine consumption differ regarding the number of prescriptions (and repeats) issued for that medicine, the quantity (by weight or count), or cost. For example, high local costs might limit the use of a medicine in a county where people have to pay for medicines “out of pocket”. Physiological differences among ethnic groups could affect the doses prescribed, and distribution and storage issues could affect pack sizes. The DDD metric (also sometimes referred to as “consumption”) overcomes these differences in prescribing trends and provides an estimate of medicine use. DDDs are allocated by the WHO Collaborating Centre working with the WHO International Working Group on Drug Statistics Methodology, [11] and only one DDD is assigned for each ATC code and route of administration. 

Although most substances have an assigned ATC code, some do not have a DDD assigned and these include: Topical products and antineoplastic agents, vaccines and sera, anesthetics, as well as allergen extracts and contrast media. Most ophthamologicals (S01) and otologicals (S02) do not have assigned DDDs, although some antiglaucoma drugs are an exception [11].

## 4. Expressing DDD Use

DDD use is a measure and should not be confused with a dose. Medicine use in the general population is usually expressed as DDD per 1000 inhabitants per day or year [11]. For instance, a value of 10 DDD/1000/day reflects an average use of 10 DDD per 1000 inhabitants on any given day of the year; i.e., 1% of the population take the standard dose (DDD) each day, or as 2% of the population taking 0.5 DDD each day. This type of information is particularly useful for medicines which are prescribed long-term to manage chronic conditions, and where the DDD is close to the PDD [8].

Medicine use in a facility such as a hospital is often expressed as DDD per bed days or DDD per 100 bed days, to reflect inpatient use. Although there is no official standardized definition of a bed day it usually reflects a patient who is confined to bed and remains in the facility overnight. As more patients undergo medical procedures or surgery as “day” patients, the definition will need to be clarified. 

### 4.1. Applications of the ATC/DDD Methodology

The methodology is a tool that can be applied widely and underpins vital knowledge about medicine use. Pharmacoepidemiological studies using the ATC/DDD methodology provide valid and consistent comparisons of medicine use within and across countries to support better outcomes and quality use of medicines [9]. For example, if there is high use of an antibiotic in region X, without a valid therapeutic reason, it could reflect indiscriminate prescribing. Similarly, use can be compared between health facilities such as hospitals and clinics. Given the threat of antimicrobial resistance, calculating comparative antibiotic use is of regional, national, and international significance. A recent example is a drug utilization study to investigate the use of quinolones associated with the treatment and or prophylaxis of COVID-19 [16]. Calculating DDDs per bed days can provide benchmarking data for in-hospital drug use; for example, 70 DDDs per 100 bed days of hypnotics estimates that 70% of the inpatients receive one DDD of a hypnotic every day; alternatively, every inpatient received 0.7 DDDs [8].

Studies on medicines inform the development of essential medicine lists (EML) [17] and standard treatment guidelines (STG) [18], and are directly relevant to policy and resource allocation. Knowing the use of medicines assists procurement and payers about the availability and cost-effective use of medicines. The methodology used to determine medicine use underpins drug utilization studies. For example, a study from a tertiary care hospital in India described a system for undertaking antimicrobial stewardship with a mechanism for prospective audit [19] and a study from the UK developed indicators for monitoring antibiotic use [20].

Medicine use is an important outcome to measure the impact of interventions and regulatory or policy actions, particularly with health and medicine policies. More countries are adopting the ATC within their own classification systems of medicinal products as the ATC/DDD classification can be consistently used by all stakeholders in the medicine chain, i.e., manufacturers, wholesalers, insurance payers, pharmacies, and regulators [8].

### 4.2. Sources of Data

The sources of data on medicine use are varied and can be grouped into three broad categories, namely, when medicines are procured, dispensed, or consumed. Data on medicine use are often collected routinely, including data generated from claims databases, or data may be purposively collected to meet specific objectives. Data may be collected at various levels of the health system, e.g., a local health facility or a national claims database. Procurement or sales data may be obtained from stakeholders in the supply chain including importers, manufacturers, wholesalers, and retailers at various geographical levels. Much of these data may have been acquired by commercial companies such as IQVIA (iqvia.com accessed 16 March 2021) and are often difficult to access without substantial funds. 

The data of dispensed use of medicines can be the most useful source for medicine use studies. Such data are routinely collected and stored in electronic databases. Reimbursement or “claims” data from a national health insurance system offer the most comprehensive data with the option to consider use aggregated at the national level down or at individual use via a unique identifier. Some of the data that are publicly available at an aggregated level will only be accessible to those within government organizations. It is hoped that as LMICs develop their UHC systems, they consider making their claims data available for *bona fide* research partners with appropriate access. Claims data from private health insurance companies are often linked to the appointment or employment of personnel at various organizations and could prove to be a useful source particularly if it were aggregated across several companies. This would complement the data from public insurance agencies especially as they are likely to reflect different socio-economic populations. Private hospitals (including those affiliated with religious institutions) and health facilities at all levels of a health system [21] that are not covered by public health insurance, as well as community pharmacies, are valuable sources of data but access may be restricted to employees. These databases may include information on both patient characteristics (e.g., age, gender, location) and medicines characteristics (dose, duration of treatment, indication, and co-prescribing).

Other sources of data include prescribing (but not the actual dispensing) of medicines; such data may become more readily available through patient encounters with prescribers based in the community who generate electronic prescriptions and electronic medical records. Individual surveys of patients may also provide vital data on actual consumption, although these are costly to produce and maintain. It is important that one considers access to all medicine use data in the light of privacy concerns, especially of individual level data. Ethical approval of studies, secure and reliable electronic storage, use by approved persons, and possible costs to obtain data are other considerations for drug utilization or pharmacoepidemiological studies. 

### 4.3. Calculating Dispensed Use

Once we have the data on the number of dispensed medicines, we can calculate the dispensed use (e.g., DDD/1000 persons) of single-ingredient medicines in a specific time period (e.g., days) by dividing the amount of drug (e.g., in milligrams) by the product of the DDD, number of inhabitants, and time period. This is calculated as follows:

*[Prescriptions × Mass × Quantity × 1000]/[DDD × Population × Time], which can be expressed as an equation:*

(1) $$DDD\;per\;1000\;persons\;per\;day=\;\frac{N\times M\times Q\times 1000}{DDD\times P\times T}$$

where

- Prescriptions refers to the number of prescriptions generated or dispensed (N);
- Mass is the dose in, e.g., milligrams or grams (M);
- Quantity refers to the pack size (Q);
- DDD is the figure assigned in the WHO guidelines (check dose units);
- Population is the sample size reflected (P); the calculation is multiplied by 1000 to convert the population size to “per 1000 population”;
- Time is the number of days of the study duration (days).

We can illustrate the calculation using an example of domperidone (21,200 prescriptions of 10 mg tablets packed in containers of 25 tablets each) in the month of June, by a population of 21 million inhabitants:

$$\begin{array}{l} \frac{21,200\;prescriptions\;\times \;10\;mg\;\times \;25\;\times \;1000}{30\;mg\;\times \;21,000,000\;\times \;30\;days}\\ =0.28\;DDDs/1000\;inhabitants/per\;day,\\ or\;0.28\;DDDs\;domperidone\;use\;by\;1000\;people\;in\;a\;day. \end{array}$$

## 5. Special Considerations (Alterations, Combinations)

Improved adherence has been associated with reduced pill burden, and the number of fixed dose combination products (FDC) has increased in recent years [11]. The main principle for calculating the use of fixed dose combination products is to count the combination as a single dose, regardless of the number of active ingredients in the combination. Medicine shortages or unavailability of fixed dose combination products in LMIC may necessitate the use of free-equivalent combinations, which are individual products that equate to the same dose if the patient had been able to obtain or purchase the fixed dose combination product. For example, a patient might use the angiotensin receptor blocker candesartan with hydrochlorothiazide in a single tablet or capsule as a fixed dose combination product, or the patient may need to take a tablet or capsule containing candesartan and a tablet or capsule containing hydrochlorothiazide as free-equivalent combinations. Fixed dose combination products are classified according to their main indication; for example, if a combination product contains an analgesic and a sedative and is indicated for the treatment of pain, the ATC will reflect analgesic. If the same analgesic were included in an antispasmodic combination product it would be classified as a gastrointestinal product. In some cases, there is a fifth-level ATC classification for combination products, e.g., N02BE51 paracetamol, and combinations excluding psycholeptics [11]. In recent years, as the number of combination products has increased, separate third or fourth ATC levels have been assigned to combinations, e.g., C10B lipid modifying agents, combinations, J05AR antivirals for treatment of HIV infections, combinations, N02AJ opioids in combination with non-opioid analgesics, and R03AL adrenergics in combination with anticholinergics including triple combinations with corticosteroids.

It is recommended that any fixed dose combination products that are included in drug utilization studies are described in detail, with the necessary rigor applied to sourcing the appropriate classification. 

There may be some confusion when calculating the use of fixed dose combination products, so we have given “normal” examples of antihypertensives for a fixed dose combination and a fixed-equivalent combination. An example of a combination that is not “normal” includes amoxicillin/clavulanic acid, which is discussed below. The key variables when calculating use are the DDD and the dose of the active ingredient(s). This case study draws on WHO guidelines to calculate medicine use, using the DDD per 1000 inhabitants (or population) per day measure for a single and fixed combination product [11].

In this section, we use the example of amlodipine and valsartan as single ingredient products, respectively, followed by a consumption calculation for the combination product amlodipine plus valsartan. 

### 5.1. Single Ingredient—Amlodipine 5 mg

Using a case study of 1500 prescriptions in a single year of 5 mg amlodipine (supplied in packs of 28 tablets) to a population of 30,000 people, the consumption is calculated at 3.84 DDD per 1000 inhabitants per day:

$$\begin{array}{l} \frac{Prescriptions\;\times \;Quantity\;\times \;1000\;inhabitants}{Dosing\;interval\;per\;day\;\times \;Population\;\times \;Time}\\ \;=\frac{1500\;\times \;5\;mg\;\times \;28\;tablets\;\times \;1000}{5\;mg\;DDD\;\times \;30,000\;\times \;365\;days}\\ \;=\frac{1500\;\times \;5\;\times \;28\;\times \;1000}{5\;\times \;30,000\;\times \;365}\\ =3.836\;DDDs\;per\;1000\;inhabitants\;per\;day\;in\;one\;year \end{array}$$

### 5.2. Single Ingredient—Valsartan 80 mg 

The calculation for single ingredient valsartan follows similar stages and for the same variables above, the consumption is also 3.836 DDDs per 1000 inhabitants per day in one year:

$$\begin{array}{l} \frac{1500\;\times \;80\;mg\;\times \;28\;\times \;1000}{80\;mg\;DDD\;\times \;30,000\;\times \;365}\\ =3.836\;DDD/1000/day \end{array}$$

### 5.3. Fixed Dose Once-Daily Amlodipine Plus Valsartan Antihypertensive

One would find that the use of fixed dose combination product of amlodipine plus valsartan would follow the same pattern if the variables were the same, i.e., 1500 prescriptions for 30,000 inhabitants in a one-year period. The use of the FDC product would also be 3.836 DDDs per 1000 inhabitants per day over a one-year period. 

Thus, it can be seen in the above example that use of a fixed dose combination antihypertensive with a once-daily dosing was the same as the use of the single FDC products; it is not the sum of the consumption of each ingredient. 

The examples given here are not the only methods that can be used to determine use of FDCs; for example, one tablet could be assigned 1 DDD for that fixed combination, or the use of the main ingredient could be calculated. For other combination products such as the antimicrobial amoxicillin plus clavulanic acid, which is included to maintain the stability and function of amoxicillin, the DDD should be equal to that of the main active ingredient, i.e., amoxicillin. Researchers should be mindful when calculating medicine use metrics for fixed dose combination products and consult the WHO source for any updates [11].

## 6. Conducting a Study

When planning a drug utilization study, one should clearly define the research question(s) and objectives regardless of whether this is a one-off research study, or it will form the basis of an ongoing monitoring program. The researcher then needs to decide on the best available data source to answer the question(s), and pragmatically tailor the choice of data source(s) to the question [8], seeking ethical approval and any other permission as appropriate, and sourcing funds if this is required. The data sources are increasing within LMIC and it is often advisable to join communities of practice [4] to garner ideas and approaches. Whatever the data source, the main steps will be to link the medicines data to the ATC codes, apply the DDD formula to calculate medicine use, and interpret patterns within the context of patients, regulations and policies, and health systems [22,23]. We recommend the use of the RECORD-PE initiative [24], which focuses on methods for doing pharmacoepidemiological research and evaluating the quality of published papers.

## 7. Conclusions

We provided a summary of the main considerations when doing a medicine use study using ATC/DDD methodology; more information is available elsewhere [8,11]. 

We encourage readers to publish their studies in academic literature, although we caution about predatory journals [25] for there are two issues of potential concern: quality control via the peer review process, and dissemination to the scientific and clinical community. 

## Data Availability

Not applicable.
